# Supplementary material for: Carbon hollow fiber membranes for a molecular sieve with precise-cutoff ultramicropores for superior hydrogen separation
Source: Nat Commun. 2021 Jan 11;12:268. doi: 10.1038/s41467-020-20628-9 (PMC7801458; doi:10.1038/s41467-020-20628-9)
Supplement: Supplementary file 1 — Supplementary Information [file 41467_2020_20628_MOESM1_ESM.pdf]

Supplementary Information for

Carbon hollow fiber membranes for a molecular sieve with precise-cutoff ultramicropores for superior hydrogen separation

Linfeng Lei[1], Fengjiao Pan[2], Arne Lindbråthen[1], Xiangping Zhang[2], Magne Hillestad[1], Yi Nie[2], Lu Bai[2], Xuezhong He[1,3*], Michael D. Guiver[4*]

Affiliations

[1]Department of Chemical Engineering, Norwegian University of Science and Technology, NO-7491 Trondheim, Norway.

[2]Beijing Key Laboratory of Ionic Liquids Clean Process, Institute of Process Engineering, Chinese Academy of Sciences, P.O. Box 353, Beijing 100190, China.

[3]Department of Chemical Engineering, Guangdong Technion Israel Institute of Technology (GTIIT), 241 Daxue Road, Shantou 515063, China.

[4]State Key Laboratory of Engines, School of Mechanical Engineering, and Collaborative Innovation Center of Chemical Science and Engineering (Tianjin), Tianjin University, Tianjin 300072, China.

*Corresponding author email: xuezhong.he@gtiit.edu.cn (X. He); michael.guiver@outlook.com (M. D. Guiver)

Table of contents

Supplementary Note 1. Tuning coagulation temperature.

Supplementary Note 2. Preparation of asymmetric cellulose hollow fiber membrane.

Supplementary Note 3. Carbonization protocols and proposed carbonization mechanism for cellulose.

Supplementary Note 4. Nanoindentation test

Supplementary Note 5. XPS and Raman characterization.

Supplementary Note 6. Apparent activation energies for the CHFMs.

Supplementary Note 7. Possible issues for membrane fabrication scale-up

Supplementary Fig. 1. Comparative cross-sectional SEM images of flat sheet membranes cast at various coagulation bath temperatures.

Supplementary Fig. 2. SEM cross-sectional image of a flat-sheet cellulose dense membrane cast at T_c 25 °C and T_d 60 °C.

Supplementary Fig. 3. Comparative cross-sectional SEM images of flat-sheet membranes cast with various thicknesses. a) the whole thickness is the selective layer; b) and c) asymmetric films with dense selective layers and porous support layers.

Supplementary Fig. 4. Cross-sectional SEM images of a cellulose hollow fiber carbon membrane precursor with ambient air drying, directly from water-wetted membranes.

Supplementary Fig. 5. Cross-sectional SEM images (a and b) of a cellulose hollow fiber precursor dried after anti-collapse treatment. c) FTIR analysis of the dried cellulose fibers. d) TGA analysis of cellulose hollow fiber.

Supplementary Fig. 6. Carbonization protocols for cellulose hollow fiber precursors.

Supplementary Fig. 7. Load–displacement curves of CHFMs from nanoindentation tests.

Supplementary Fig. 8. Pore size distribution of carbon films, calculated by the NLDFT model from CO_2 physisorption at 0 °C.

Supplementary Fig. 9. Surface area and pore volume of CHFMs

Supplementary Fig. 10. CO_2 uptake of CHFMs

Supplementary Fig. 11. a) XPS survey spectra, and b) O 1s spectra for three different CHFMs

Supplementary Fig. 12. a) C 1s XPS spectra and b) Raman spectra of three different CHFMs.

Supplementary Fig. 13. A Proposed mechanism of transformation from cellulose precursors to CMS membranes.

Supplementary Fig. 14. The evolved small gas molecules of CO_2, H_2, H_2O, and CH_4 from cellulose carbonization under different carbonization procedures as measured by TGA-MS.

Supplementary Fig. 15. Arrhenius plots for H_2 and CO_2 permeances, a) CHFM-550. b) CHFM-700 and c) CHFM-850.

Supplementary Fig. 16. Normalized H_2 permeance (a) and H_2/CO_2 selectivity (b) as a function of aging time, tested at 130 °C for single gas.

Supplementary Fig. 17. Illustration of the high-pressure mixed gas permeation rig with humidity control

Supplementary Fig. 18. A representative module used for mixed gas permeation measurements operated in a counter-current flow pattern.

Supplementary Fig. 19. Contact angles of water on the CMS membranes prepared at different carbonization temperatures

Supplementary Fig. 20. Comparison of separation performance of CHFMs with state-of-the-art inorganic membrane materials on an upper bound plot presenting gas permeance (GPU).

Supplementary Fig. 21. Comparison of separation performance of CHFM-850 with state-of-the-art inorganic membrane materials. a) H_2/N_2 separation. b) H_2/CH_4 separation.

Supplementary Fig. 22. Schematic of membrane module for carbon hollow fiber membranes, a) for mixed gas testing, and b) for single gas testing.

Supplementary Fig. 23. Photographs of a membrane module being bent with a dimeter of 3.7 cm and used for single gas permeation tests.

Supplementary Table 1. Cellulose hollow fiber spinning conditions.

Supplementary Table 2. Hardness, reduced modulus, and Young's modulus of CHFMs

Supplementary Table 3. Elemental composition of the CHFMs from XPS analysis.

Supplementary Table 4. Membrane performances of polymeric and inorganic membranes presented in Fig. 4.

Supplementary Table 5. Membrane performances of inorganic-based membranes presented in Supplementary Fig. 21a.

Supplementary Table 6. Membrane performances of inorganic-based membranes presented in Supplementary Fig. 21b.

Supplementary Note 1. Tuning coagulation temperature

Temperatures of the dope solutions (T_d) and coagulation bath (T_c) were investigated to determine the optimal membrane formation conditions. Different flat sheet membranes were cast under various T_c conditions in the range of 25 °C to 60 °C, while T_d was maintained at 25 °C. A solvent exchange protocol was used to prevent pore morphology collapse. The water-wetted cellulose membranes were immersed into pure isopropanol for 2 h, followed by soaking in n-hexane for 2 h, and then all the membranes were allowed to dry under ambient conditions in air. It was found that the cellulose membranes had relatively dense morphology when the T_c was below 45 °C (**Supplementary Figs. 1a-c**). However, when the T_cs were ≥ 45 °C, clear asymmetric structures with a dense top layers and porous support structures were generated (**Supplementary Figs. 1d-f**). In contrast, a warmer dope solution (T_d = 60 °C) cast onto a glass plate and coagulated in a 25 °C water bath presents a dense symmetric membrane morphology, as shown in **Supplementary Fig. 2**. Thus, the coagulation bath temperature plays a crucial role for generating asymmetric cellulose membrane morphology. The critical temperature to generate asymmetric morphology is found to be ~45 °C, and asymmetric membranes could be obtained only when T_c ≥ 45 °C. Moreover, in order to obtain a relatively thinner selective layer, a T_c of 60 °C was selected for spinning cellulose hollow fibers in the subsequent work.

Flat-sheet cellulose films with different thicknesses were cast and dried under the same conditions as those used for the spinning process, as shown in **Supplementary Fig. 3**. When a thin film with a thickness of smaller than 10 μm was made (**Supplementary Fig. 3a**), the bulk film presented entirely dense morphology, which is representative of the selective layer of the CHFMs. Thus, the thinnest cellulose films were carbonized, and the obtained carbon films were used for structural characterization.

Supplementary Note 2: preparation of asymmetric cellulose hollow fiber membranes

Supplementary Fig. 4 shows cross-sectional SEM images of a cellulose hollow fiber that was dried directly in air without solvent exchange treatment to prevent pore collapse. It presents a dense and symmetric structure. However, **Supplementary Figs. 5a** and **5b** show the cross-sectional SEM images of the spun hollow fibers, and a clear asymmetric structure with a porous inner support layer and dense outer layer is evident. The asymmetric morphological structure was maintained by applying a solvent exchange protocol using first isopropanol, followed by *n*-hexane). The precursor fibers were dried in air before conducting the carbonization process. The dried cellulose hollow fibers were analyzed by FTIR and TGA, and the results are shown in **Supplementary Fig. 5c** and **5d**, respectively.

Supplementary Note 3. Carbonization protocols and proposed carbonization mechanism for cellulose

The carbonization protocols selected are based on the TGA analysis of cellulose precursors in **Supplementary Fig. 5d**. A dwell-time of 2 h at 300 ℃ was employed to take into account the significant weight loss at this temperature due cellulose depolymerization. Three types of carbon membranes were obtained by carbonization protocols at different final temperatures of 550, 700 and 850 ℃, while all other carbonization parameters (e.g., heating rate, dwell time, etc.) were the same.

Supplementary Fig. 13 outlines the transformation mechanism from cellulose precursors to CMS membranes, based on the characterization results of HR-TEM, XPS, Raman spectra and TGA-MS. When the final carbonization temperature is below 600 °C (in this work, 550 °C was used), disordered carbon "plates" were formed by intramolecular rearrangement, and a higher content of oxygen heteroatom existed in the carbon matrix by the formation of –OH, –COO, and $–CH_3$ groups, which contributed to the more disordered structure (**Supplementary Fig. 13c**). As the final carbonization temperature was increased to over 600 °C, and especially over 800 °C, pendant groups, such as –OH and $–CH_3$, were removed by forming H_2O and CO_2, which resulted in a more ordered carbon structure (**Supplementary Fig. 13d**). This was also supported by the higher sp^2 carbon content and lower oxygen content in the XPS spectra. Furthermore, according to HR-TEM and PSD, it can be proposed that the ultramicropores are from the inter-planar spacing, while the micropore contribution is from the imperfect packing of the carbon sheets.

Supplementary Note 4. Nanoindentation test

The hardness, the reduced elastic modulus, and Young's modulus of CHFMs were measured by nanoindentation tests using a Berkovich indenter. The CHFM samples were loaded to the maximum load (*Pmax* = 1 mN) in 5 s and then held for 2 s, followed by unloading in 5 s. The measured hardness and reduced elastic modulus are summarized in **Supplementary Table 2.** The Young's modulus (E, GPa) were estimated using the Oliver–Pharr method [1] as follows:

$$\frac{1}{E_r} = \frac{1-\nu^2}{E} + \frac{1-\nu_{\mathrm{tip}}^2}{E_{\mathrm{tip}}} \qquad (1)$$

Where ν and are E Poisson's ratio and Young's modulus of the CHFM samples, respectively. ν_{tip} and E_{tip} are Poisson's ratio and Young's modulus of the indenter, respectively. $\nu_{\mathrm{tip}} = 0.07$ and $E_{\mathrm{tip}} = 1140$ GPa. Poisson's ratio of CHFMs is assumed to be the same and equal to 0.2.[2] Since $E_{\mathrm{tip}} \gg E_r$, the second term of the equation S1 is negligible. Hence, the Young's modulus of the samples is approximated to $E = 0.96\ E\mathrm{r}$.

Supplementary Note 5. XPS and Raman characterization

The CHFMs were characterized by XPS and Raman spectroscopy. The carbon content increases with the increase of carbonization temperature. Also, when the carbonization temperature is increased from 550 to 850 °C, the O contents was reduced from 9.26 Atomic % to 7.04 Atomic %. The trace amount of N-element (ca. 0.6 Atomic % in CHFMs) presented in CHFMs is probably derived from residual EmimAc in the cellulose precursors. Such a low content may not have a significant effect on the micropore structure, as most of the reported N-doped porous carbon materials contain 3%-10%. **Supplementary Fig. 12a** shows each C 1s spectrum deconvoluted into three main peaks. The peak located at ~284.7 eV corresponds to sp^2 -hybridized carbon, while the peak at ~ 285.2 eV corresponds to sp^3-hybridized carbon. The third peak at ~ 286.3 eV is assigned to C−N or C−O bonds [3,4]. The fourth peak with much lower intensity located at ~ 289.2 eV is attributed to the C=O bond [3,4].

The Raman spectra of the CHFMs were obtained by deconvolution of the spectra into 5 peaks - D1, D2, D3, D4 and G (**Supplementary Fig. 12b**). All CHFMs exhibit two major peaks, namely the G peak (Graphite band), located at ~1600 cm^{-1}, which corresponds to the E_{2g}-symmetry vibration mode of sp^2 hybridized carbon, and the D1 peak (Defect band) located at ~1346 cm^{-1} which is the A_{1g}-symmetry vibration mode from the disordered graphite [5,6]. The D1 band is active when ring defects (ring breaks) are present within the graphite planes. The D2 band at ~ 1620 cm^{-3} is assigned to graphitic lattice vibrations mode with E_{2g} symmetry (disordered graphitic lattice). The D3 and D4 band are generally exhibited in highly disordered carbonaceous materials [5,6]. The D3 band, located at ~1525 cm^{-1} is usually ascribed to amorphous carbon, while the D4 band at ~1165 cm^{-1} is attributed to disordered graphitic lattice or sp^3 impurities [7].

Supplementary Note 6. Apparent activation energies for the CHFMs

The apparent activation energies for the CHFMs were calculated by the Arrhenius relationship between gas permeance and testing temperature. Gas permeability (*P*, barrer) is the product of diffusivity (*D*) and sorption coefficient (*S*). Thus, gas permeance (*P/l*, GPU) can be described by Supplementary Equation (2),

$$\frac{P}{l} = \frac{D \times S}{l} \tag{2}$$

where l (µm) is the membrane thickness of selective layer. For carbon molecular sieve (CMS) membranes, the diffusivity and solubility coefficients can be described by the Arrhenius equation (Supplementary Equation (3)) and the Van't Hoff equation (Supplementary Equation (4)), respectively [8]:

$$D = D_0 e^{-\frac{E_D}{RT}} \tag{3}$$

$$S = S_0 e^{-\frac{H_S}{RT}} \tag{4}$$

Where D_0 and S_0 are the pre-exponential factor for diffusion and sorption, respectively. E_D and H_S are the apparent diffusion activation energy and the apparent heat of sorption, respectively. Thus, the gas permeance in Supplementary Equation (2) can be rewritten as,

$$\frac{P}{l} = \frac{P_0}{l} e^{-\frac{E_P}{RT}} \tag{5}$$

Where $E_P = E_D + H_S$, is the apparent activation energy. $P_0 = D_0 \times S_0$. The E_P can be calculated by linear regression of the ln (*P/l*) versus 1/T, namely by Supplementary Equation (6):

$$\ln\left(\frac{P}{l}\right) = \ln\left(\frac{P_0}{l}\right) - \frac{E_P}{R}\frac{1}{T} \tag{6}$$

The apparent activation energies of for both H_2 and CO_2 permeate through different CHFMs are presented in **Supplementary Fig. 15**.

Supplementary Note 7. Possible issues for membrane fabrication scale-up

Scaling-up of CHFMs prepared from cellulose and ionic liquids may still face the following challenges, and the potential solutions are listed correspondingly.

1. Reducing the ratio of EmimAc/DMSO in dope solution and recovering EmimAc can be applied to bring down the relatively high cost of ionic liquids.
2. It is crucial to drain the tars and remove vapors during the carbonization if large amounts of fibers are carbonized in a furnace. By setting a small angle (e.g., 6°) between the quartz support and furnace can be used for draining tars.
3. Membrane module design and construction are also important, such as CHFM mounting, potting and sealing. Due to the H_2/CO_2 separation are often used under high -temperature and -pressure conditions, a better potting material (compared to epoxy resin used in this work) that can endure the humidified gas at high pressure and temperature should be identified.

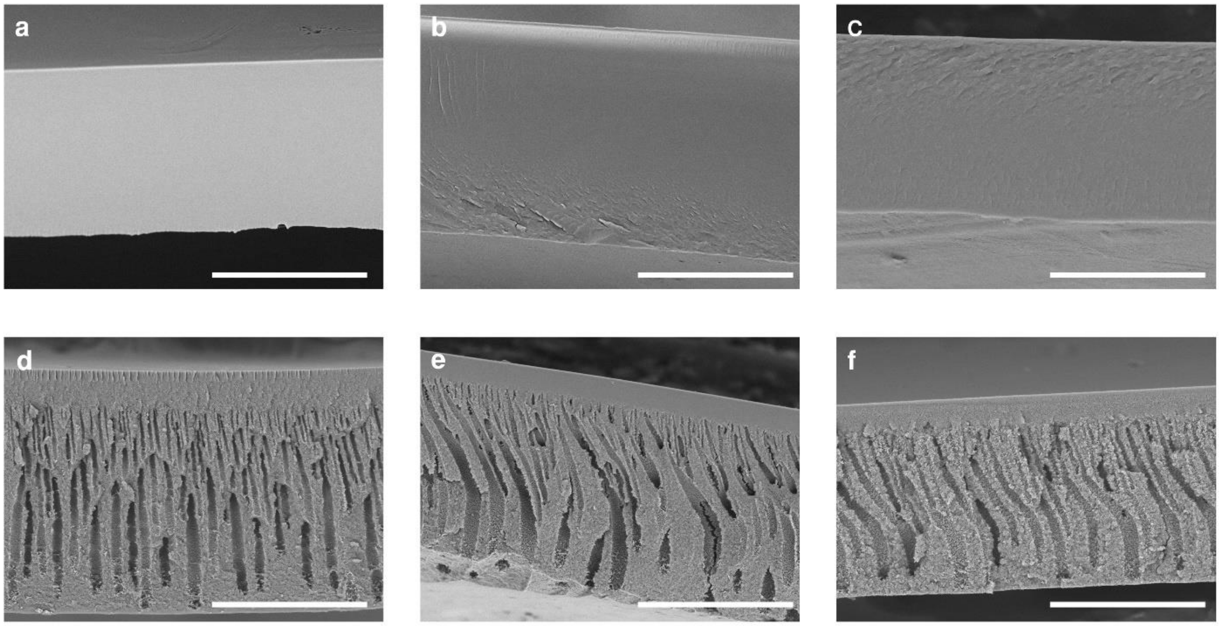

Supplementary Fig. 1. Comparative cross-sectional SEM images of flat sheet membranes cast at various coagulation bath temperatures (T_c). a) 25 °C, b) 35 °C, c) 40 °C, d) 45 °C, e) 50 °C and f) 60 °C. The dope solution temperature (T_d) was maintained at 25 °C. Scale bars: 100 µm.

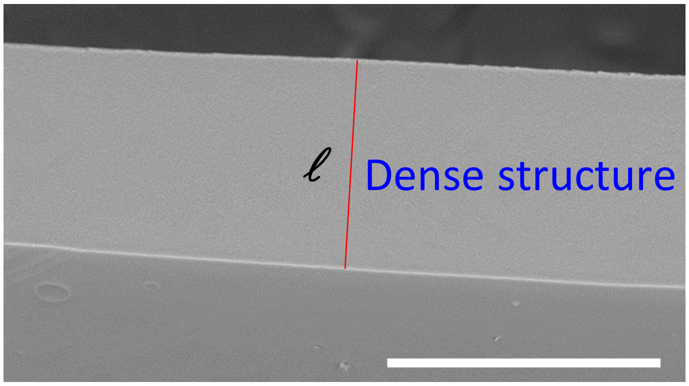

Supplementary Fig. 2. SEM cross-sectional image of a flat-sheet cellulose dense membrane cast at T_c 25 °C and T_d 60 °C. Scale bar: 100 µm.

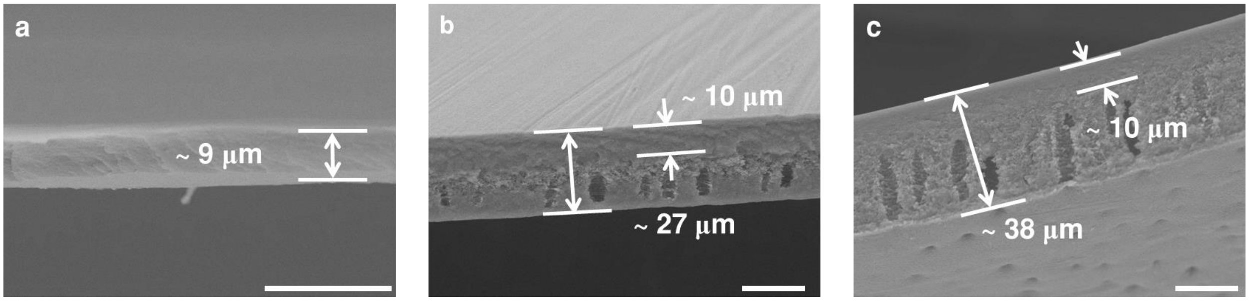

Supplementary Fig. 3. Comparative cross-sectional SEM images of flat-sheet membranes cast with various thicknesses. a) the whole thickness is the selective layer; b) and c) asymmetric films with dense selective layer and porous support layer. Scale bar: 20 μm.

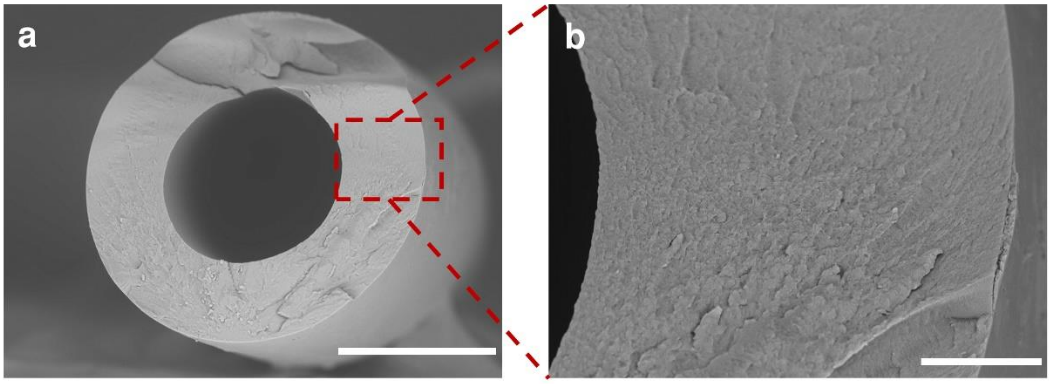

Supplementary Fig. 4. Cross-sectional SEM images of a cellulose hollow fiber carbon membrane precursor with ambient air drying, directly from water-wetted membranes. The hollow fiber presents a symmetric structure. Scale bars: a-200 μm, b-30 μm.

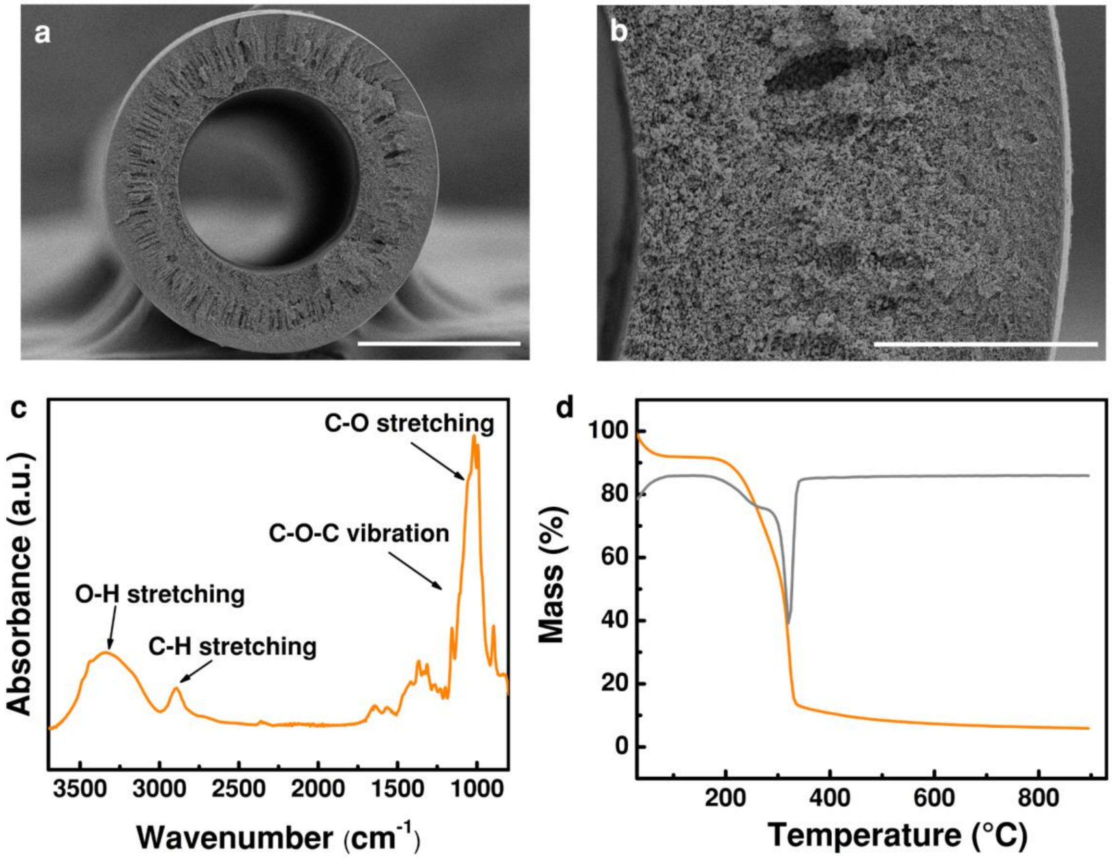

Supplementary Fig. 5. Cross-sectional SEM images (a and b) of a cellulose hollow fiber precursor dried after anti-collapse treatment. The hollow fiber presents an asymmetric structure with a dense outer layer and a porous inner support. Scale bars: a-200 μm, b-50 μm. c) FTIR analysis of the dried cellulose fibers. d) TGA analysis of cellulose hollow fiber. A significant weight loss occurs in the temperature range of 280-330 °C.

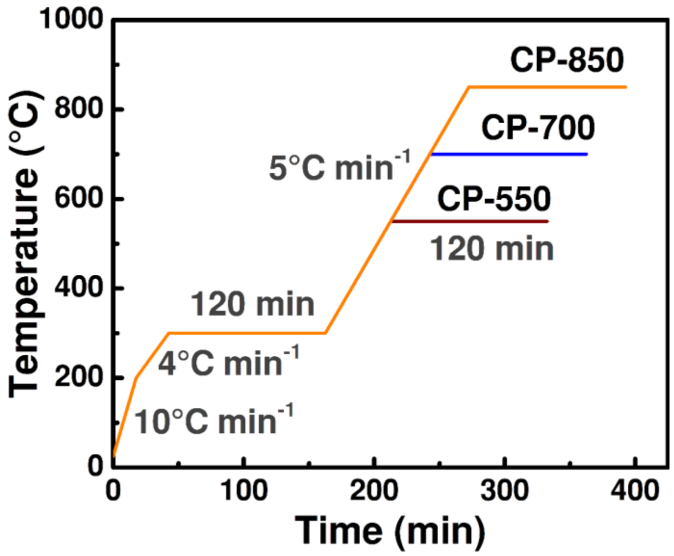

Supplementary Fig. 6. Carbonization protocols for cellulose hollow fiber precursors conducted in argon atmosphere at a continuous flow of 80 mL min^{-1} with different final carbonization temperatures varying from 550-850 °C.

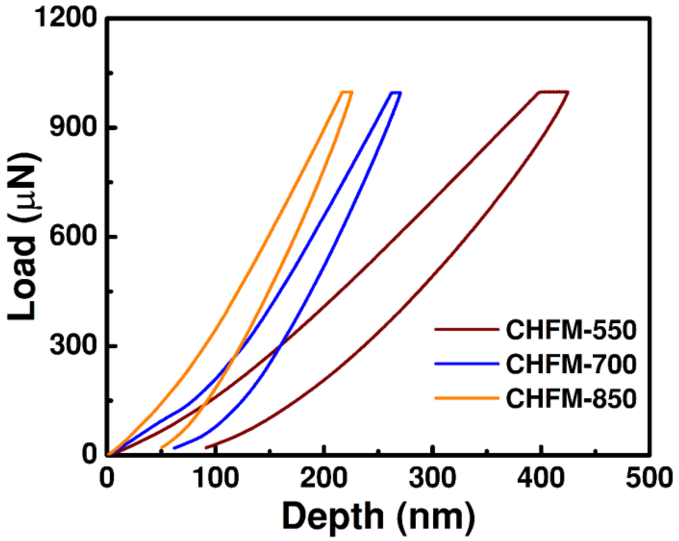

Supplementary Fig. 7. Load–displacement curves of CHFMs from nanoindentation tests.

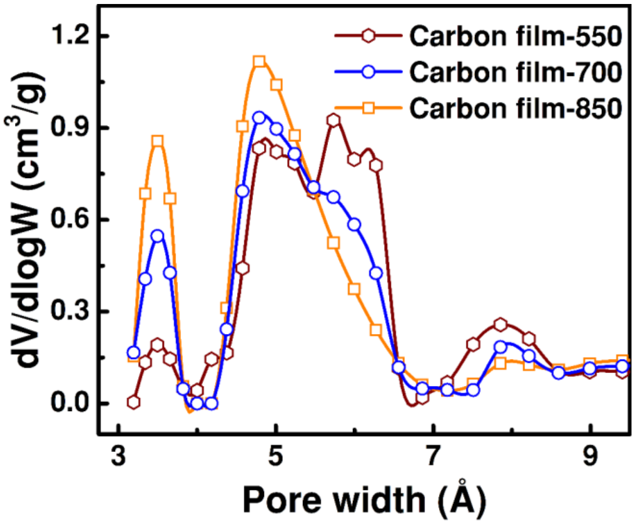

Supplementary Fig. 8. Pore size distribution of carbon films, calculated by the NLDFT model from CO_2 physisorption at 0 °C.

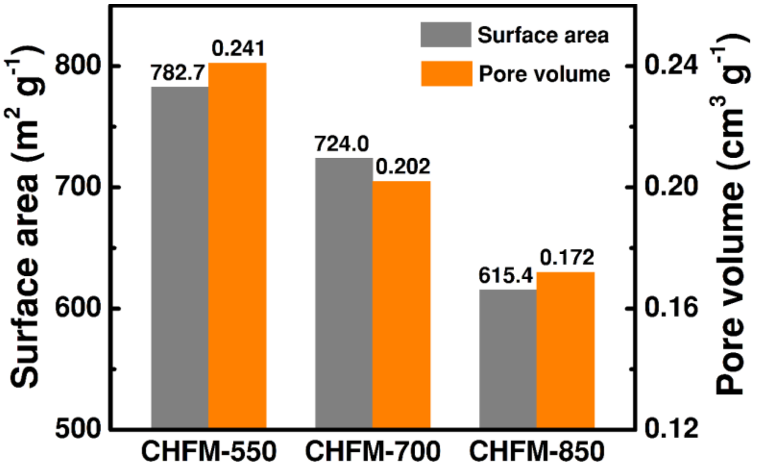

Supplementary Fig. 9. The surface area and pore volume of CHFMs calculated by the NLDFT model from CO_2 physisorption at 0 °C.

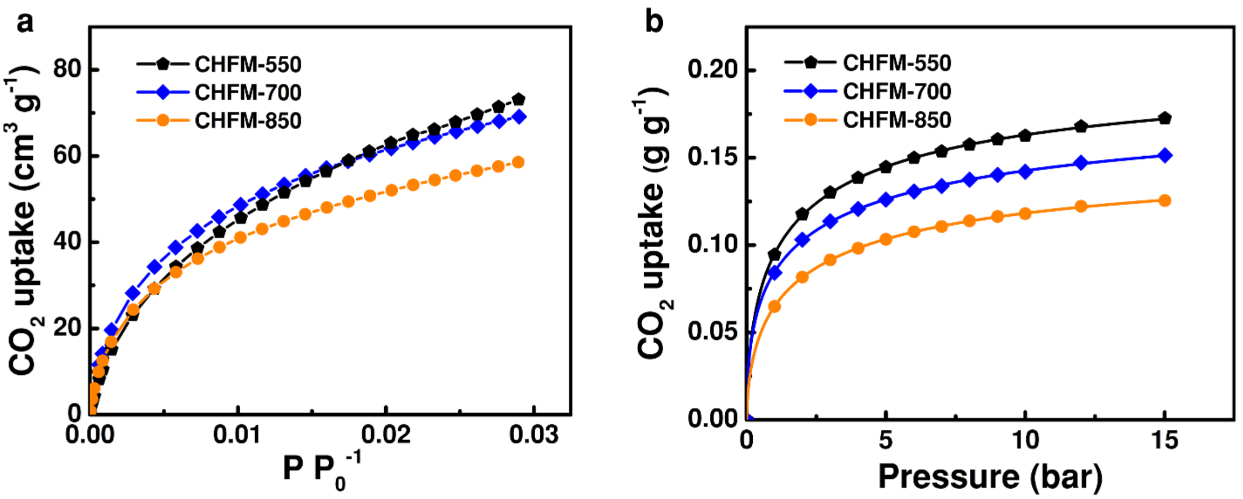

Supplementary Fig. 10. CO_2 uptake amount of CHFMs. a) low pressure CO_2 sorption obtained at 0 °C from pressure range of 0-1 bar. b) high pressure CO_2 sorption at 25 °C ranging from 1-15 bar (carried out by a Rubotherm equipped with a magnetic balance).

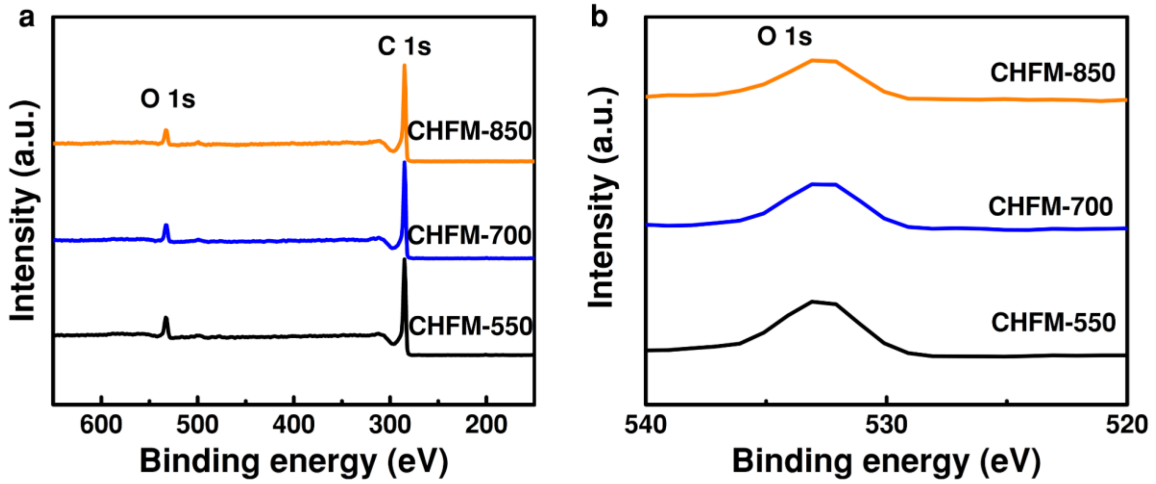

Supplementary Fig. 11. a) XPS survey spectra, and b) O 1s spectra for three different CHFMs

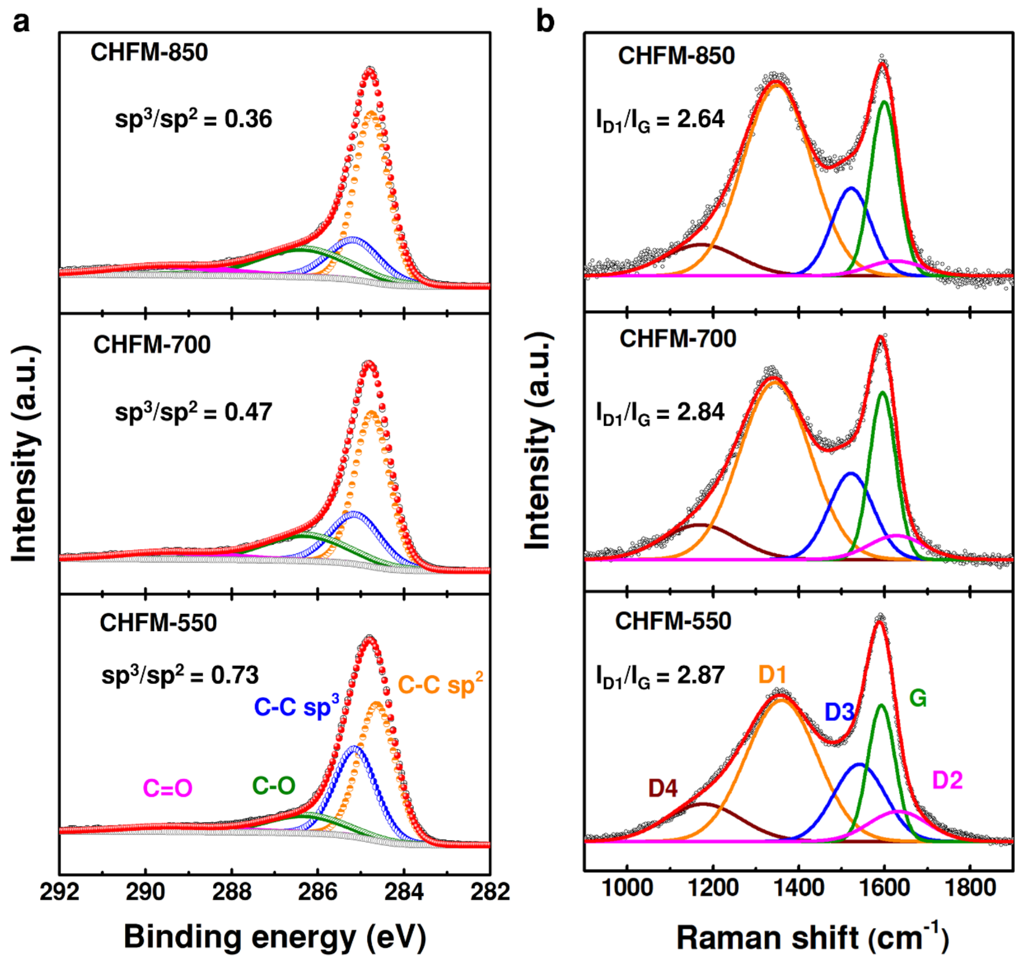

Supplementary Fig. 12. a) C1s XPS spectra of the CHFM samples, the transformation of sp^3 to sp^2-hybridized carbon as the ratios of sp^3 and sp^2 hybridized carbon decrease from 0.73-0.36 following the carbonization temperature from 550 to 850 °C; b) Raman spectra of CHFM samples. The prominent peaks of G and D1 correspond to the E_{2g}-symmetry vibration mode of sp^2 hybridized carbon and the A_{1g}-symmetry vibration mode from disordered graphite, respectively. The D2 band at ~ 1620 cm^{-1} is assigned to graphitic lattice vibrations mode with E_{2g}-symmetry (disordered graphitic lattice). The D3 band, located at ~1525 cm^{-1} is usually ascribed to amorphous carbon, while the D4 band at ~1165 cm^{-1} is attributed to disordered graphitic lattice or sp^3 impurities.

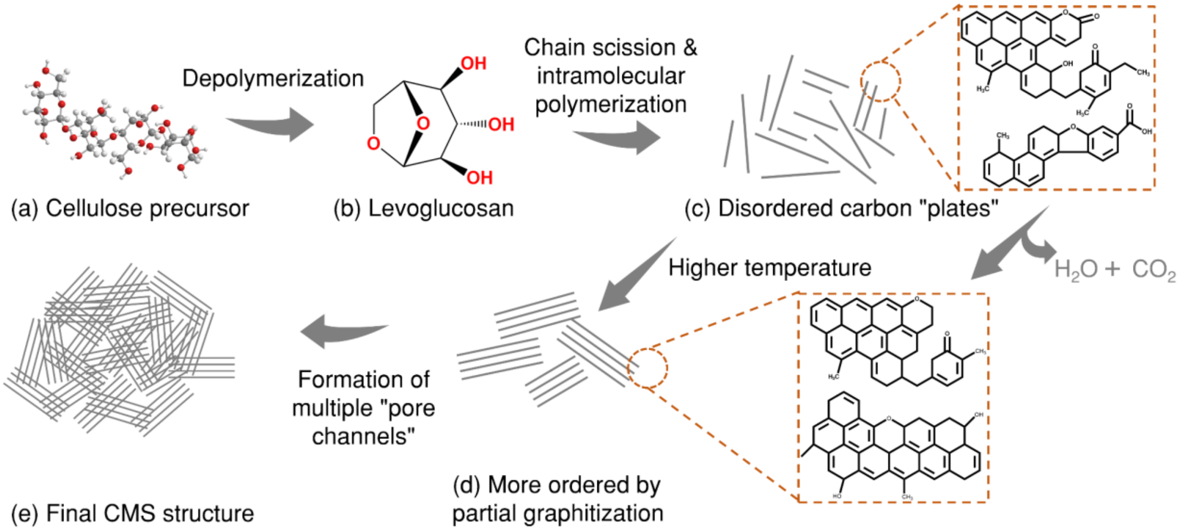

Supplementary Fig. 13. A Proposed mechanism of transformation from cellulose precursors to CMS membranes.

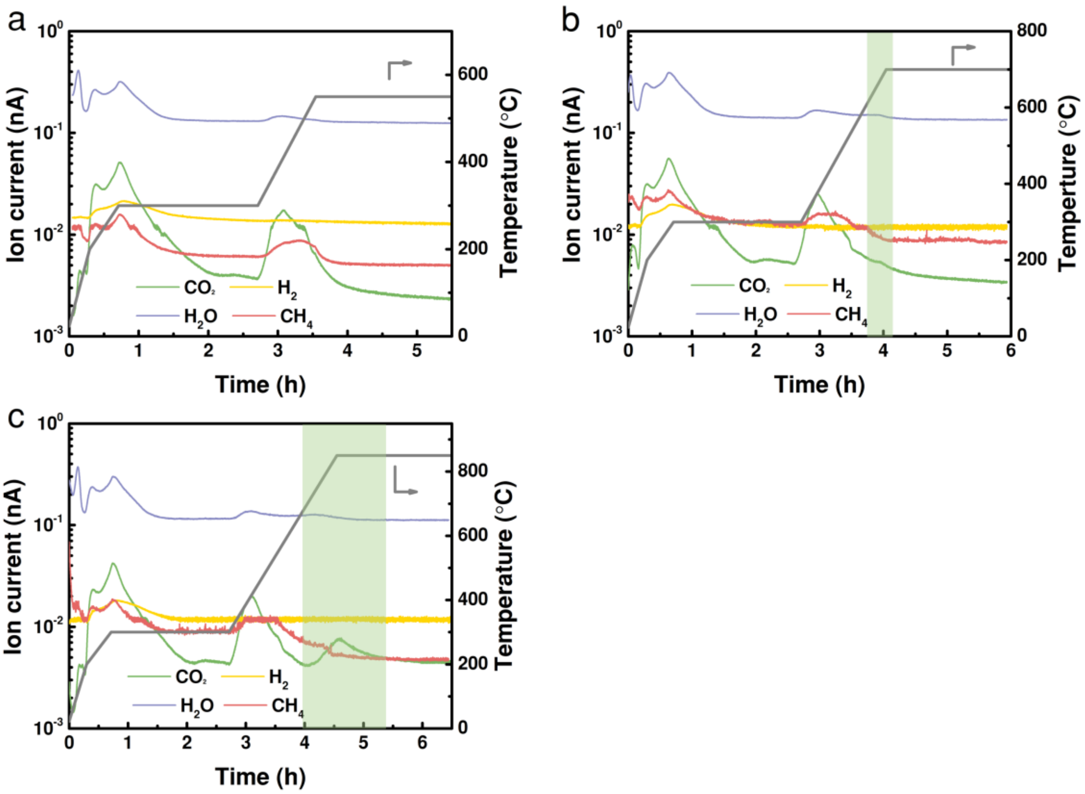

Supplementary Fig. 14. The evolved small gas molecules CO_2, H_2, H_2O, and CH_4 from cellulose carbonization under different carbonization procedures as measured by TGA-MS.

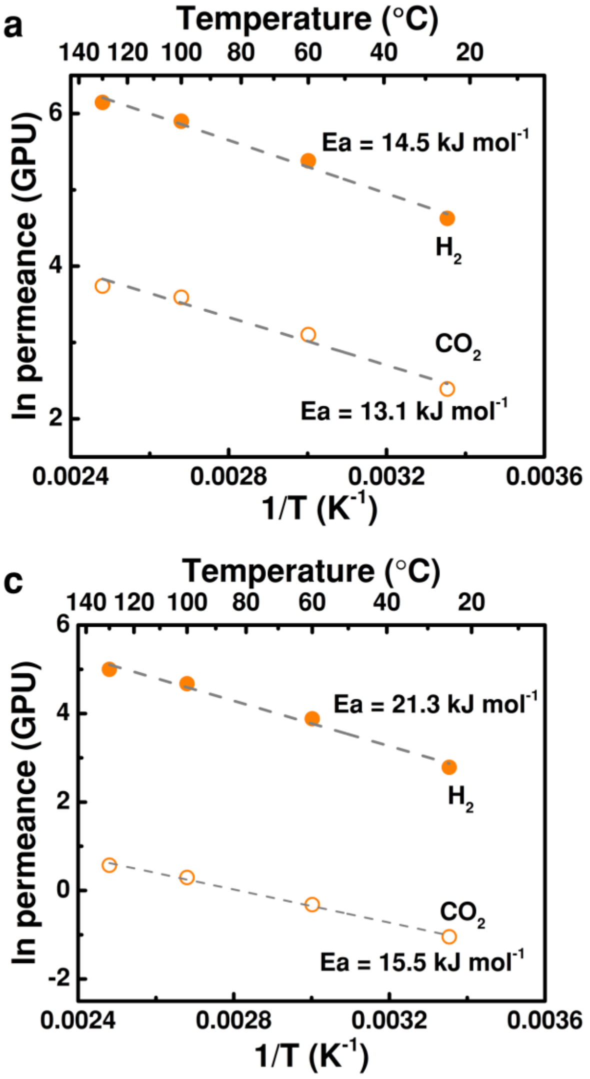

Supplementary Fig. 15. Arrhenius plots for H_2 and CO_2 permeances, a) CHFM-550. b) CHFM-700 and c) CHFM-850.

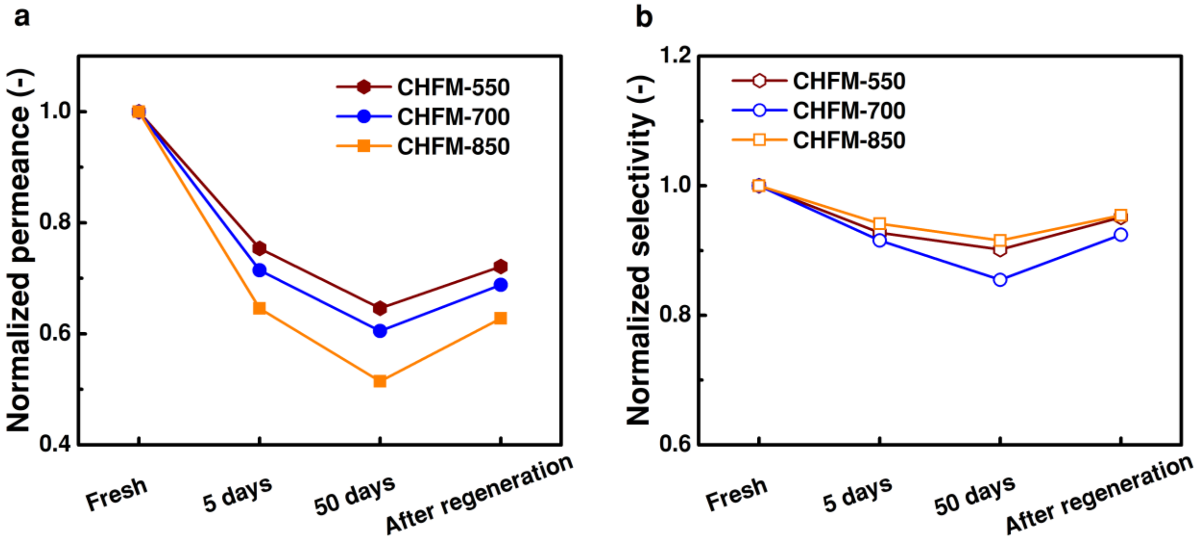

Supplementary Fig. 16. Normalized H_2 permeance (a) and H_2/CO_2 selectivity (b) as a function of aging time, tested at 130 °C for single gas.

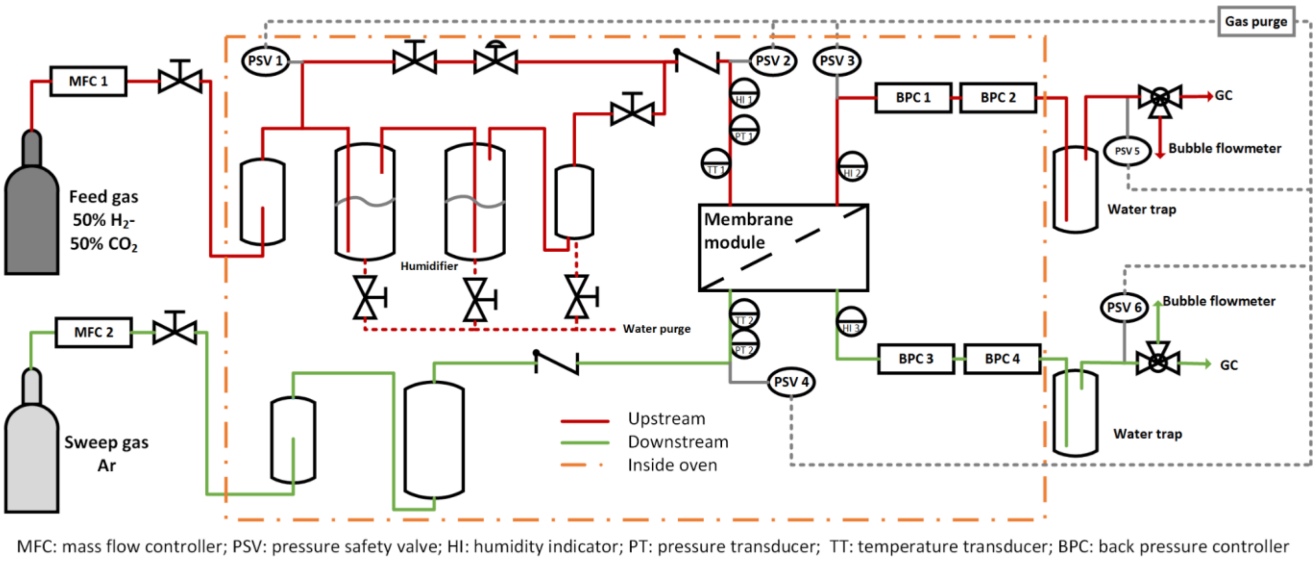

Supplementary Fig. 17. Illustration of the high-pressure mixed gas permeation rig with humidity control

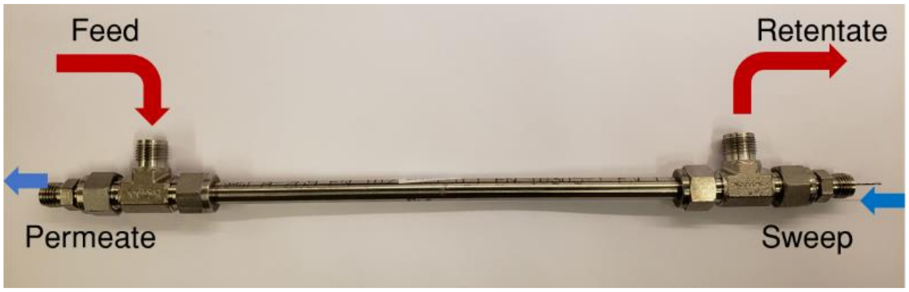

Supplementary Fig. 18. A representative module used for mixed gas permeation measurements operated in a counter-current flow pattern. Gas is fed from the shell side, and the permeate gas comes from the bore side using argon as sweep gas.

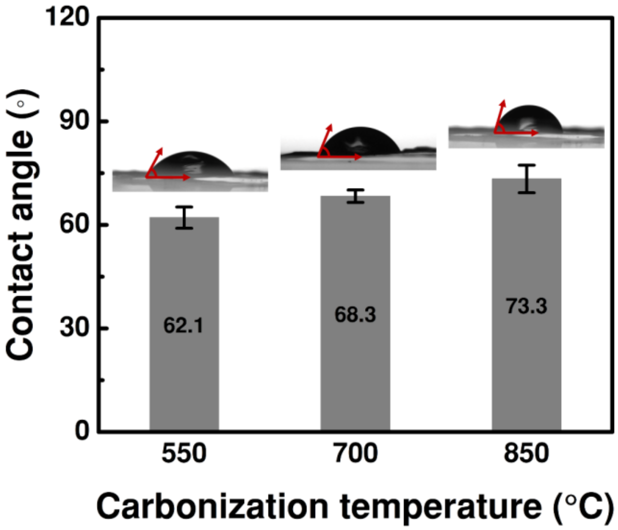

Supplementary Fig. 19. Contact angles of water on the CMS membranes prepared at different carbonization temperatures

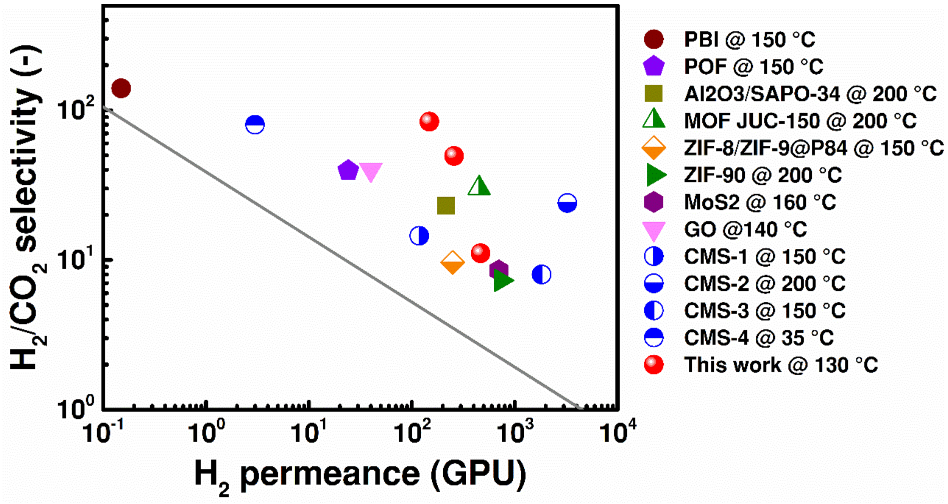

Supplementary Fig. 20. Comparison of separation performance of CHFMs with state-of-the-art inorganic membrane materials on an upper bound plot presenting gas permeance (GPU). The solid line is based on the 2008 Robeson upper bound line [9] by converting permeability to permeance (assuming a membrane thickness of 1 μm).

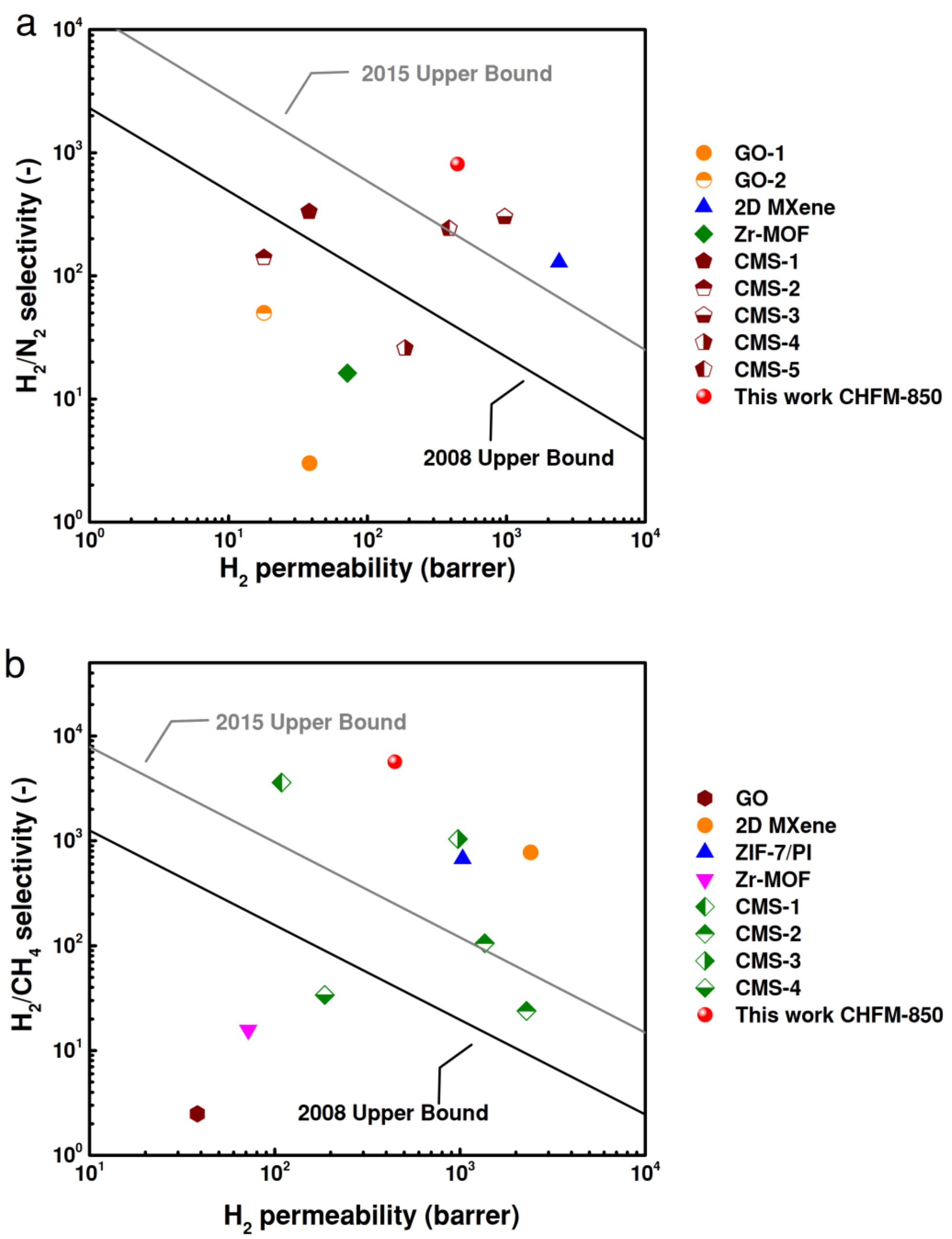

Supplementary Fig. 21. Comparison of separation performance of CHFM-850 with state-of-the-art inorganic membrane materials. a) H_2/N_2 separation. b) H_2/CH_4 separation. Details of the comparative membranes are listed in **Supplementary Tables 5** and **6**. The black and gray lines are based on the 2008 Robeson upper bound [9] and 2015 upper bound [10], respectively.

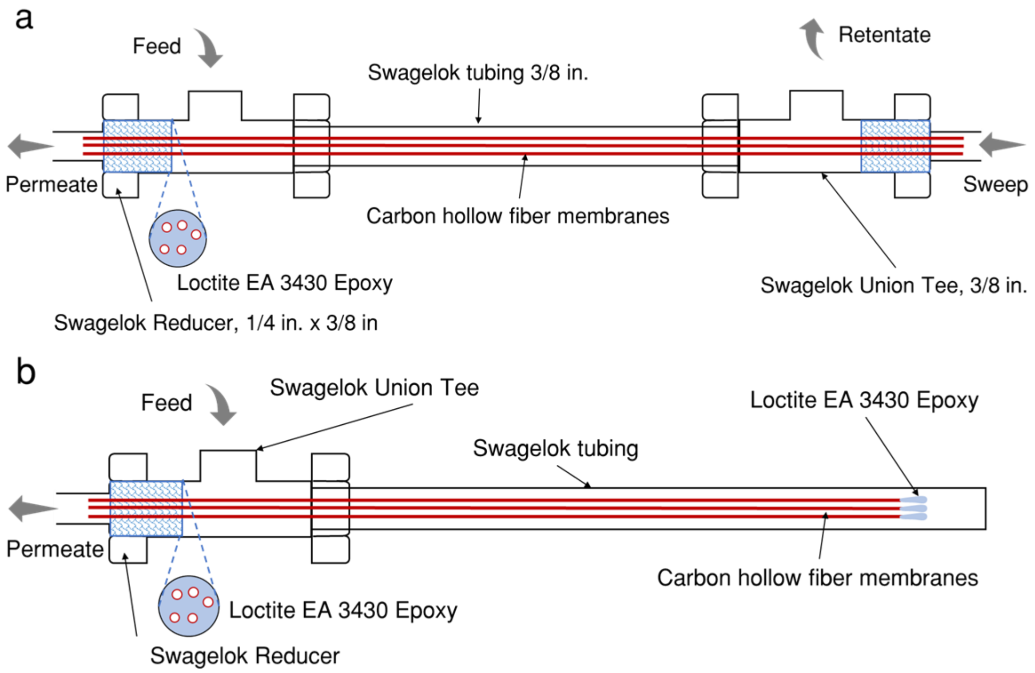

Supplementary Fig. 22. Schematic of membrane module for carbon hollow fiber membranes, a) for mixed gas testing, and b) for single gas testing.

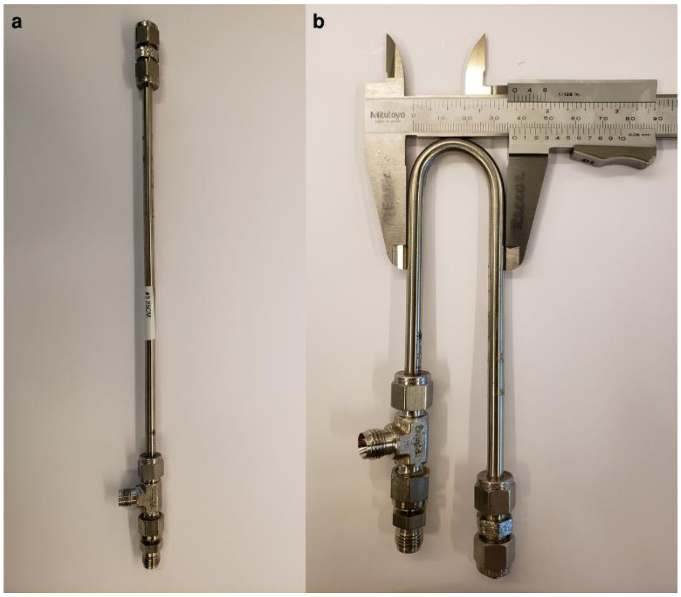

Supplementary Fig. 23. Photographs of a membrane module being bent with a dimeter of 3.7 cm and used for single gas permeation tests.

Supplementary Table 1. Cellulose hollow fiber spinning conditions.

Spinning conditions	Value
Dope solution composition and temperature	12% MCC in (75 wt.% EmimAc + 25 wt.% DMSO), 25 °C
Bore fluid composition and temperature	20% Water in (75 wt.% EmimAc + 25 wt.% DMSO), 25 °C
First coagulation bath temperature	60 °C
Second coagulation bath temperature	40 °C
Dope flow rate	4.4 mL min^{-1}
Bore flow rate	1.8 mL min^{-1}
Take up speed	14.6 m min^{-1}
Air gap	8 cm
Spinneret OD/ID	0.7/0.5 mm

Supplementary Table 2. Hardness, reduced modulus, and Young's modulus of CHFMs

Samples	Hardness (GPa)	Reduced modulus (GPa)	Young's modulus (GPa)
CHFM-550	0.31±0.06	2.16±0.26	2.07±0.25
CHFM-700	0.84±0.25	5.93±0.06	5.69±0.07
CHFM-850	1.30±0.10	7.85±0.50	7.53±0.48

Supplementary Table 3. Elemental composition of the CHFMs from XPS analysis.

	C (Atomic %)	O (Atomic %)	N (Atomic %)
CHFM-550	90.08	9.26	0.67
CHFM-700	91.25	8.10	0.65
CHFM-850	92.41	7.04	0.55

Supplementary Table 4. Membrane performances of polymeric and inorganic membranes presented in Fig. 4. The reported permeances (GPU) in literature were converted to permeability (barrer) based on selective layer thickness of membranes (1 μm was assumed if the thickness is not given).

Membrane materials	Performance		Test conditions			Reference
	H_2 Permeability (barrer)	α_{H_2/CO_2}	Type of analysis	Temp. (°C)	Feed pressure (bar)	
PBI	1.5	140	Single gas	150	14	11
POF	24.2	39.5	Mixed gas	150	2	12
MOF JUC-150	452.4 (based on 1 μm)	30.2	Mixed gas	200	1	13
Al_2O_3/SAPO-34	214.7 (based on 1 μm)	23.0	Mixed gas	200	3.5	14
ZIF-8/ZIF-9@P84	500	9.6	Mixed gas	150	-	15
ZIF-90	14784	7.3	Single gas	200	1	16
GO	~40	~40	Single gas	140	1	17
MoS_2	240	8.5	Mixed gas	160	1	18
CMS-1	1065	14.5	Single gas	150	-	19
CMS-2	650	24	Single gas	200	2	7
CMS-3	228.4	8	Single gas	150	1	20
CMS-4	54	80	Single gas	150	11	21
CHFM-550	1400.3	11.1	Single gas	130	2	This work
CHFM-700	773.3	49.5	Single gas	130	2	This work
CHFM-850	444.6	83.9	Single gas	130	2	This work

Supplementary Table 5. Membrane performances of inorganic-based membranes presented in Supplementary Fig. 21a. The separation performances, which were reported as permeance (GPU) in literature, were converted to permeability (barrer).

Membrane materials	Performance		Test conditions			Reference
	H_2 Permeability (barrer)	α_{H_2/N_2}	Type of analysis	Temp. (°C)	Feed pressure (bar)	
CMS-1	38.1	331	Single gas	22	-	[22]
CMS-2	18.0	141	Mixed gas	220	2	[23]
CMS-3	974	302	Single gas	21	6	[24]
CMS-4	186.3	25.8	Single gas	21	-	[25]
CMS-5	388.3	243	Single gas	25	1	[26]
GO-1	38.2	~3	Single gas	25	1	[17]
GO-2	~18	~50	Mixed gas	100	1	[27]
2D MXene	2402	129	Single gas	25	1	[28]
Zr-MOF	71.9	16.2	Single gas	25	1	[29]
This work CHFM-850	444.6	829.0	Single gas	130	2	

Supplementary Table 6. Membrane performances of inorganic-based membranes presented in Supplementary Fig. 21b.

Membrane materials	Performance		Test conditions			Reference
	H_2 Permeability (barrer)	α_{H_2/CH_4}	Type of analysis	Temp. (°C)	Feed pressure (bar)	
CMS-1	108.6	3620	Single gas	29.5	2	30
CMS-2	1359	106	Single gas	130	1.5	31
	2283	24.1	Single gas	130	1.5	
CMS-3	974	1047	Single gas	21	6	24
CMS-4	186.3	34.2	Single gas	21	-	25
ZIF-7@polyimide	674	128.4	Single gas	100	2	32
GO	38.2	2.5	Single gas	25	1	17
2D MXene	2402	780	Single gas	25	1	27
Zr-MOF	71.9	15.7	Single gas	25	1	29
This work CHFM-850	444.6	5706	Single gas	130	2	

References

1. Oliver WC, Pharr GM. An improved technique for determining hardness and elastic modulus using load and displacement sensing indentation experiments. *Journal of Materials Research* **7**, 1564-1583 (1992).

2. McCartney LN. Predicting Properties of Undamaged and Damaged Carbon Fibre Reinforced Composites. In: *The Structural Integrity of Carbon Fiber Composites: Fifty Years of Progress and Achievement of the Science, Development, and Applications* (Springer International Publishing, 2017).

3. Niu J, *et al.* Biomass-derived mesopore-dominant porous carbons with large specific surface area and high defect density as high performance electrode materials for Li-ion batteries and supercapacitors. *Nano Energy* **36**, 322-330 (2017).

4. Lee SW, *et al.* High-power lithium batteries from functionalized carbon-nanotube electrodes. *Nature Nanotechnology* **5**, 531-537 (2010).

5. Sadezky A, Muckenhuber H, Grothe H, Niessner R, Pöschl U. Raman microspectroscopy of soot and related carbonaceous materials: Spectral analysis and structural information. *Carbon* **43**, 1731-1742 (2005).

6. Gao J, *et al.* Construction of a sp^3/sp^2 Carbon Interface in 3D N-Doped Nanocarbons for the Oxygen Reduction Reaction. *Angewandte Chemie International Edition* **58**, 15089-15097 (2019).

7. Ngamou PHT, Ivanova ME, Guillon O, Meulenberg WA. High-performance carbon molecular sieve membranes for hydrogen purification and pervaporation dehydration of organic solvents. *Journal of Materials Chemistry A* **7**, 7082-7091 (2019).

8. Rungta M, *et al.* Carbon molecular sieve structure development and membrane performance relationships. *Carbon* **115**, 237-248 (2017).

9. Robeson LM. The upper bound revisited. *Journal of Membrane Science* **320**, 390-400 (2008).

10. Swaidan R, Ghanem B, Pinnau I. Fine-Tuned Intrinsically Ultramicroporous Polymers Redefine the Permeability/Selectivity Upper Bounds of Membrane-Based Air and Hydrogen Separations. *ACS Macro Letters* **4**, 947-951 (2015).

11. Zhu L, Swihart MT, Lin H. Unprecedented size-sieving ability in polybenzimidazole doped with polyprotic acids for membrane H_2/CO_2 separation. *Energy & Environmental Science* **11**, 94-100 (2018).

12. Shan M, *et al.* Facile manufacture of porous organic framework membranes for precombustion CO_2 capture. *Science Advances* **4**, eaau1698 (2018).

13. Kang Z, *et al.* Highly selective sieving of small gas molecules by using an ultra-microporous metal–organic framework membrane. *Energy & Environmental Science* **7**, 4053-4060 (2014).

14. Yu M, Funke HH, Noble RD, Falconer JL. H_2 Separation Using Defect-Free, Inorganic Composite Membranes. *Journal of the American Chemical Society* **133**, 1748-1750 (2011).

15. Cacho-Bailo F, *et al.* On the molecular mechanisms for the H_2/CO_2 separation performance of zeolite imidazolate framework two-layered membranes. *Chemical Science* **8**, 325-333 (2017).

16. Huang A, Dou W, Caro J. Steam-Stable Zeolitic Imidazolate Framework ZIF-90 Membrane with Hydrogen Selectivity through Covalent Functionalization. *Journal of the American Chemical Society* **132**, 15562-15564 (2010).

17. Kim HW, *et al.* Selective Gas Transport Through Few-Layered Graphene and Graphene Oxide Membranes. *Science* **342**, 91 (2013).

18. Achari A, S S, Eswaramoorthy M. High performance MoS_2 membranes: effects of thermally driven phase transition on CO_2 separation efficiency. *Energy & Environmental Science* **9**, 1224-1228 (2016).

19. Sá S, Sousa JM, Mendes A. Steam reforming of methanol over a $CuO/ZnO/Al_2O_3$ catalyst part II: A carbon membrane reactor. *Chemical Engineering Science* **66**, 5523-5530 (2011).

20. Richter H, *et al.* High-Flux Carbon Molecular Sieve Membranes for Gas Separation. *Angewandte Chemie International Edition* **56**, 7760-7763 (2017).

21. Omidvar M*, et al.* Unexpectedly Strong Size-Sieving Ability in Carbonized Polybenzimidazole for Membrane H_2/CO_2 Separation. *ACS Applied Materials & Interfaces* **11**, 47365-47372 (2019).

22. Shiflett MB, Foley HC. Ultrasonic Deposition of High-Selectivity Nanoporous Carbon Membranes. *Science* **285**, 1902 (1999).

23. Hou J*, et al.* Carbon Nanotube Networks as Nanoscaffolds for Fabricating Ultrathin Carbon Molecular Sieve Membranes. *ACS Applied Materials & Interfaces* **10**, 20182-20188 (2018).

24. Ogieglo W, Puspasari T, Ma X, Pinnau I. Sub-100 nm carbon molecular sieve membranes from a polymer of intrinsic microporosity precursor: Physical aging and near-equilibrium gas separation properties. *Journal of Membrane Science* **597**, 117752 (2020).

25. Ogieglo W, Furchner A, Ma X, Hazazi K, Alhazmi AT, Pinnau I. Thin Composite Carbon Molecular Sieve Membranes from a Polymer of Intrinsic Microporosity Precursor. *ACS Applied Materials & Interfaces* **11**, 18770-18781 (2019).

26. Tseng H-H, Wang C-T, Zhuang G-L, Uchytil P, Reznickova J, Setnickova K. Enhanced H_2/CH_4 and H_2/CO_2 separation by carbon molecular sieve membrane coated on titania modified alumina support: Effects of TiO_2 intermediate layer preparation variables on interfacial adhesion. *Journal of Membrane Science* **510**, 391-404 (2016).

27. Li H*, et al.* Ultrathin, Molecular-Sieving Graphene Oxide Membranes for Selective Hydrogen Separation. *Science* **342**, 95 (2013).

28. Ding L*, et al.* MXene molecular sieving membranes for highly efficient gas separation. *Nature Communications* **9**, 155 (2018).

29. Ghalei B*, et al.* Rational Tuning of Zirconium Metal-Organic Framework Membranes for hydrogen purification. *Angewandte Chemie International Edition* **58**, 19034-19040 (2019).

30. Campo MC, Magalhães FD, Mendes A. Carbon molecular sieve membranes from cellophane paper. *Journal of Membrane Science* **350**, 180-188 (2010).

31. Huang S*, et al.* Ultrathin Carbon Molecular Sieve Films and Room-Temperature Oxygen Functionalization for Gas-Sieving. *ACS Applied Materials & Interfaces* **11**, 16729-16736 (2019).

32. Ma X, Wu X, Caro J, Huang A. Polymer Composite Membrane with Penetrating ZIF-7 Sheets Displays High Hydrogen Permselectivity. *Angewandte Chemie International Edition* **58**, 16156-16160 (2019).
